# Genomic landscape and gene expression profiles of feline oral squamous cell carcinoma

**DOI:** 10.3389/fvets.2023.1079019

**Published:** 2023-05-17

**Authors:** Alana R. Rodney, Zachary L. Skidmore, Jennifer K. Grenier, Obi L. Griffith, Andrew D. Miller, Shirley Chu, Faraz Ahmed, Jeffrey N. Bryan, Santiago Peralta, Wesley C. Warren

**Affiliations:** ^1^Department of Animal Sciences, University of Missouri, Columbia, MO, United States; ^2^McDonnell Genome Institute, Washington University School of Medicine, St Louis, MO, United States; ^3^Department of Biomedical Sciences, College of Veterinary Medicine, Cornell University, Ithaca, NY, United States; ^4^Department of Oncology, School of Veterinary Medicine, University of Missouri, Columbia, MO, United States; ^5^Department of Clinical Sciences, College of Veterinary Medicine, Cornell University, Ithaca, NY, United States

**Keywords:** whole exome sequencing, feline oral squamous cell carcinoma, human head and neck cancer, variant calling comparisons, cancer

## Abstract

Feline oral squamous cell carcinoma (FOSCC) is a cancer of the squamous cell lining in the oral cavity and represents up to 80% of all oral cancers in cats, with a poor prognosis. We have used whole exome sequencing (WES) and RNA sequencing of the tumor to discover somatic mutations and gene expression changes that may be associated with FOSCC occurrence. FOSCC offers a potential comparative model to study human head and neck squamous cell carcinoma (HNSCC) due to its similar spontaneous formation, and morphological and histological features. In this first study using WES to identify somatic mutations in feline cancer, we have identified tumor-associated gene mutations in six cats with FOSCC and found some overlap with identified recurrently mutated genes observed in HNSCC. Four samples each had mutations in *TP53*, a common mutation in all cancers, but each was unique. Mutations in other cellular growth control genes were also found such as *KAT2B* and *ARID1A*. Enrichment analysis of FOSCC gene expression profiles suggests a molecular similarity to human OSCC as well, including alterations in epithelial to mesenchymal transition and IL6/JAK/STAT pathways. In this preliminary study, we present exome and transcriptome results that further our understanding of FOSCC.

## Introduction

Feline oral squamous cell carcinoma (FOSCC) is the fourth most common cancer, and the most commonly found malignant oral tumor in cats (1), with a 1-year survival rate of < 10% (2). FOSCC arises from the normal squamous epithelium of the oral (1) (gingiva, tongue, and sublingual regions) cavity. FOSCC rarely metastasizes to distant locations; however, the lymph nodes can be affected in 13–31% of cases (3). Early studies indirectly implicated using flea collars, feeding predominantly canned foods in the diet to increase the risk of development of FOSCC up to 5.3-fold (3) as well as environmental tobacco smoke (4). There are currently no broadly effective treatment options for most cats with FOSCC. Surgical excision has shown to be an effective method to treat FOSCC and other oral tumors with cats having 1-year survival rates of over 80% (5, 6). Chemotherapy or radiation are additional alternatives, but most owners don't opt for these treatments due to side effects and for most cats, the median survival time is only 2–6 months (7, 8). FOSCC presents with similar features as HNSCC, such as inflammation, spontaneous formation, heterogeneous cell environment, natural tumor, and host immune system interactions, and thus may present an opportunity to model comparative therapeutics (9, 10).

HNSCC is the sixth most common cancer worldwide, with 550,000 new cases per year, and has a 5-year survival rate of 50% (11, 12). Like FOSCC, if HNSCC is diagnosed in the early stages survival rates are much higher (2, 13, 14). Predicted risk factors for HNSCC include exposure to tobacco smoke, alcohol, and infection with HPV (13–15). Molecular similarities between the two species have been reported but these presumptions are only based on candidate gene studies in FOSCC. Both humans and cats show the perturbed function of p53, altering cell metabolism, preventing cell cycle arrest, and apoptosis (16). Gain of function in p53 has also been observed in HNSCC and is associated with enhanced tumor progression, invasive cell growth, and metastatic potential (17). Overexpression of *EGFR* is found in 69–100% of FOSCC and 90% of HNSCC cases, driving cycle progression, and facilitating the invasion of oral tissues (10, 18–20).

Naturally occurring companion animal models of cancer are becoming integral to the understanding of tumor evolution and progression (1, 6). The mouse is the standard model, yet those models lack critical factors such as spontaneous tumor formation as a result of acquiring somatic mutations in shared habitats, progressive tumor heterogeneity, and often a cancer-conditioned immune system. Understanding the genomic environment of both cat and human OSCC will help inform future translational studies. With many unknowns in the genetics of FOSCC, we aimed to characterize the mutational and transcriptional profile of FOSCC and contrast this with HNSCC. To accomplish this, FOSCC tumor tissue and matched blood samples were used for whole exome sequencing (WES), and RNA-seq was generated on FOSCC tumor tissue and oral cavity samples from healthy cats. Somatic mutations, their corresponding contribution to the tumor mutation burden (TMB), and differentially expressed genes (DEG) were assessed to compile a preliminary report of FOSCC genetics.

## Materials and methods

### Clinical samples

**Figure 1:**
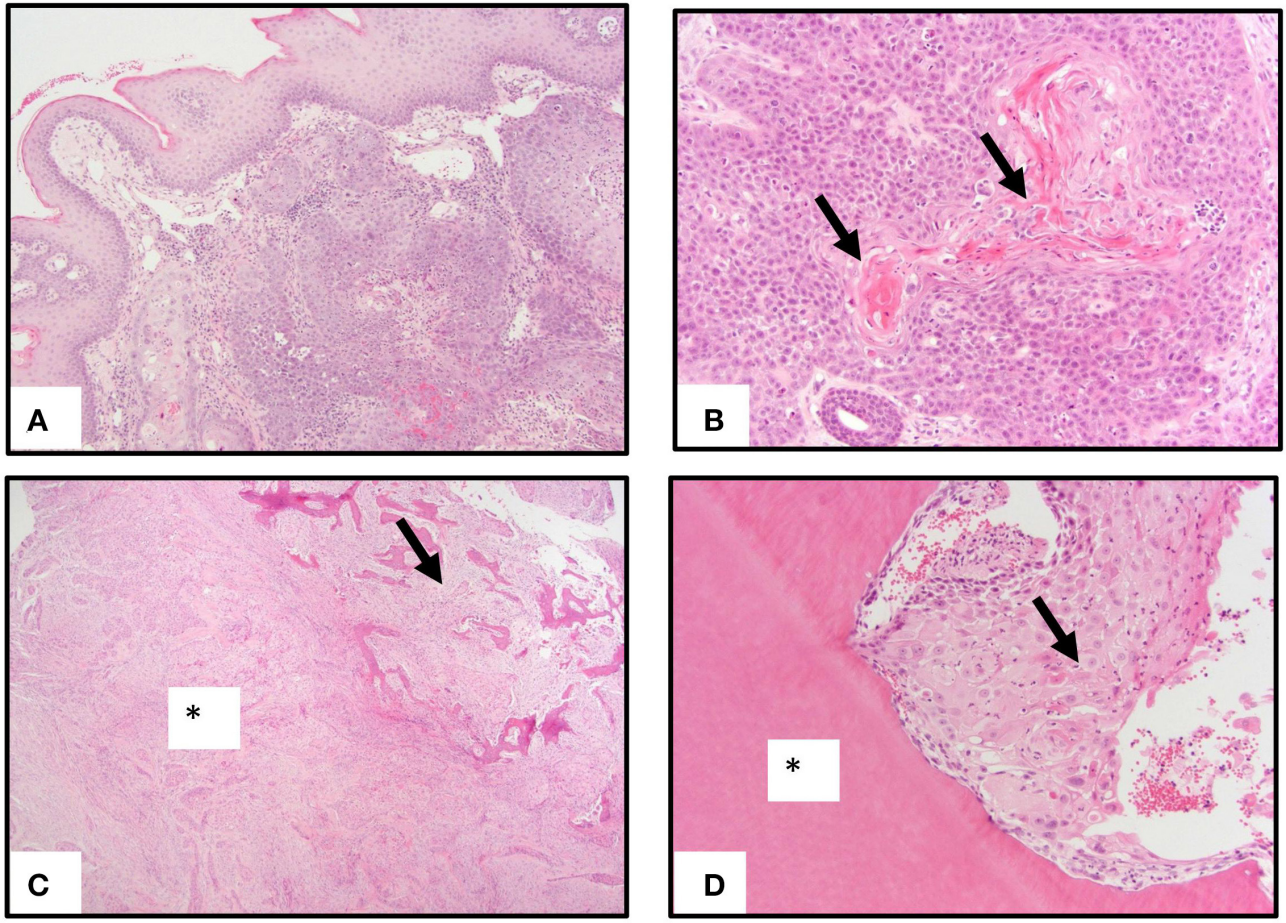
Pathology of oral squamous carcinoma. **(A)** Underlying a moderately hyperplastic gingival epithelium are ribbons, cords, and trabeculae of neoplastic squamous epithelial cells. **(B)** Neoplastic squamous epithelial cells surround and produce brightly eosinophilic keratin (arrows). **(C)** Neoplastic squamous epithelial cells that are enmeshed in abundant scirrhous responses (asterisk) are associated with marked bony invasion and remodeling (arrow). **(D)** Neoplastic squamous epithelial cells with dyskeratosis (arrow) invade the dentin layer of a tooth (asterisk).

Genomic DNA isolated from six individual FOSCC tumors and matched normal peripheral blood were used for WES; RNA isolated from four of the tumors used for WES and two additional tumors, as well as three normal oral mucosa (NOM) samples from healthy cats were used for RNA-seq (Table 1). All NOM and seven of the eight FOSCC samples came from different cats archived by the Cornell Veterinary Biobank; the FOSCC and corresponding blood samples had been collected by Cornell University Veterinary Dentistry and Oral Surgery Service during standard-of-care surgical procedures (i.e., biopsy) in accordance with IACUC approved protocol #2005-0151. One FOSCC sample (605591) was collected at the Ontario Veterinary College Companion Animal Tumor Sample Bank in accordance with IACUC approved protocol #4409. FOSCC was diagnosed by histopathology in all tumor samples by a board-certified veterinary pathologist (Figure 1).

**Table 1:** Case description table that includes detailed patients information and assays performed.

**Sample**	**Age (years)**	**Breed**	**Sex**	**Tumor/tissue location**	**Diagnosis**	**WES**	**Assays performed**	
							**RNA-seq**	**Library size (bp)**
7741	16	DSH	MC	Mandible	FOSCC	Yes	Yes	453
9895	12	DSH	FS	Mandible	FOSCC	Yes	Yes	469
19791	20	DSH	MC	Maxilla	FOSCC	No	Yes	490
24147	12	DSH	MC	Mandible	FOSCC	Yes	Yes	532
23263	13	DSH	MC	Sublingual	FOSCC	Yes	Yes	499
28139	14	DSH	FS	Maxilla	FOSCC	No	Yes	498
26903	16	DSH	MC	Mandible	FOSCC	Yes	No	N/A
605591	10	UNK	M	Mandible	FOSCC	Yes	No	N/A
23962	1	DSH	M	Maxilla	NOM	No	Yes	505
24235	1	DSH	M	Maxilla	NOM	No	Yes	559
23927	1	DSH	M	Maxilla	NOM	No	Yes	479

DSH, domestic shorthair; UNK, unknown; M, male; MC, male castrates; FOSCC, feline oral squamous cell carcinoma; NOM, normal oral mucosa; FS, male castrated; RQN, RNA quality number; bp, base pairs; N/A, not applicable.

### WES, variant calling, and annotation

WES was completed according to Rodney et al. (21). The Illumina NovaSeq6000 was used to generate paired-end 2 × 150 bp reads, producing an average of 80 × depth of sequencing coverage. Raw sequence reads were mapped to Felis_Catus_9.0 reference using Burrows-Wheeler Aligner (BWA) v0.7.17 (22) and PCR or optical duplicates were marked using Picard tools v2.19.9 (http://broadinstitute.github.io/picard/). These files were then processed through the Genome Analysis Toolkit (GATK) v4.0.1 for base quality rescore calibration. Data is available at https://www.ncbi.nlm.nih.gov/bioproject/PRJNA844099. Mutect2 was used to identify somatic single nucleotide variants (SNVs) and insertions and deletions (indels) using the main filtering parameters of *t_lod_fstar* (filters out variants with insufficient evidence of the presence in the tumor sample) and *panel_of_normals* (filters out variants present in at least two samples in the panel of normal) (Supplementary Figure S1). The generated VCF files were further cleaned for missing data and variant allele frequencies < 0.1% using VCFtools. SNVs were then annotated using Variant Effect Predictor (VEP) v101.0 (22) which classifies variants by their predicted impact on gene function. In our final call SNV and indel call set we focused on missense, frameshift (predicted changes to the amino acid), and non-sense (predicted to alter gene structure) variants. An estimation of false positives was completed using the manual viewing of IGV for a randomly chosen 20 variants per sample and confirming the presence of that variant in aligned sequences at a given base position.

### Tumor mutational burden (TMB)

TMB was calculated for all six tumor samples. For each VCF file, Felis_Catus_9.0 is set as the reference and Ensembl (release 102) was used for annotation. Starting with only non-synonymous somatic mutations we followed a similar process as reported in HNSCC studies (23, 24) (Supplementary Table S1). TMB was estimated using the number of non-synonymous SNVs divided by the total feline exon probe size of 35 MB (21), which includes non-essential splice site regions (25).

### Cross-reference of HNSCC mutated genes

Two resources of recurrently mutated genes in HNSCC were explored in this study: Driver Database version 3 (26) (DriverDBv3; accessed online on 3/15/21) and OcoKB (27) (version and access date of 3/15/21). The DriverDBv3 identified 263 genes total. For OncoKB substantially fewer genes (*n* = 10) we retrieved but this was due to filtering by druggable status. A table of all genes were collated and used for further investigation of the presence of mutations in these same genes within the FOSCC samples.

### RNA sequencing

Frozen tissue (~1 g) was homogenized in 2 mL of Trizol (Thermo Fisher) using 2.8 mm ceramic beads (Hard Tissue Homogenizing Mix, VWR). RNA was extracted from four FOSCC samples (9895, 24147, 23263, and 7741) following the Trizol protocol provided by the manufacturer (Thermo Fisher), and then treated with DNAse followed by cleanup with the RNA Clean and Concentrator-25 kit (Zymo Research). For all other samples, RNA was extracted with a modified Trizol method as follows: after the addition of chloroform and phase separation of the Trizol lysate, the aqueous phase was combined with an equal volume of 100% ethanol and loaded onto a Zymo-Spin column (Quick-RNA Prep Kit, Zymo Research). RNA samples were washed and eluted following the Quick-RNA Prep Kit protocol. For all samples, RNA concentration was measured with a Nanodrop (Thermo Fisher), and integrity was determined with a Fragment Analyzer (Agilent). Because of variable RNA quality across samples (RQN range: 1.3–8.8), whole-transcriptome RNA-seq was conducted after depleting ribosomal RNA. rRNA was depleted with the NEBNext rRNA Depletion Kit v2 (Human/Mouse/Rat; New England Biolabs) using 500 ng input total RNA. RNA-seq libraries were prepared with the NEBNext Ultra II Directional library prep kit (New England Biolabs) and single-end 85 nt reads were generated on a NextSeq500 instrument (Illumina), resulting in an average of 25.1 M reads per sample (minimum 19.1 M).

### RNA-seq analysis

Raw reads were trimmed for low-quality and adaptor sequences and filtered for minimum length with TrimGalore (http://www.bioinformatics.babraham.ac.uk/projects/trim_galore/), a wrapper for cutadapt (28) and fastQC (http://www.bioinformatics.babraham.ac.uk/projects/fastqc/) using parameters “–nextseq-trim = 20 -O 1 -a AGATCGGAAGAGC –length 50 –fastqc.” Trimmed reads were mapped to the reference genome/transcriptome (Ensembl felCat9) with STAR (29) using these parameters: “–outSAMstrandField intronMotif, –outFilterIntronMotifs RemoveNoncanonical, –outSAMtype BAM SortedByCoordinate, and –quantMode GeneCounts,” which also generated raw count outputs per annotated gene. Sample clustering and differential gene expression were analyzed with SARTools (30) and DESeq2 (31) using these parameters: “fitType parametric, cooksCutoff TRUE, independentFiltering TRUE, alpha 0.05, pAdjustMethod BH, typeTrans VST, and locfunc median.” Feline gene symbols were converted to human gene symbols using Biomart (Ensembl) one-to-one orthology assignments to enable analysis of MSigDB (32) and custom human gene sets. The human ortholog gene symbols and log2-fold-change values for expressed genes (at least one group with average normalized counts > 50) were used for GSEA (33) “Preranked” analysis. Heatmaps of leading-edge genes were generated in R (d3heatmap) using row-normalized counts.

## Results

### SNV annotation

**Figure 2:**
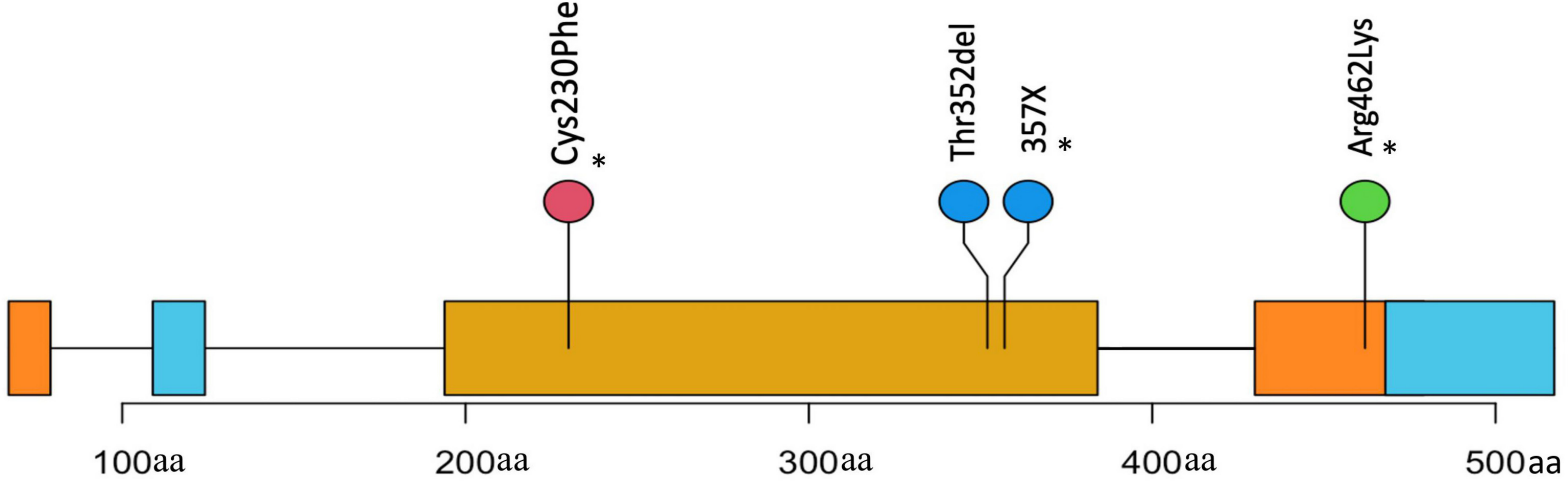
Positions and description of P53 somatic mutations in 4 samples. Three mutations(*) were missense causing an amino change in two samples and an unknown change in another (X). One mutation is a frameshift causing a deletion in the protein coding sequence.

VCF files were annotated using VEP and filtered to remove any synonymous and intronic variants. In total, we found 809 non-synonymous SNVs in the FOSCC exome with a mean of 176 per sample (Supplementary Figures S2, S3). SNV annotation with VEP resulted in 56 non-sense, 19 frameshift mutations, 8 stop loss, and 731 missense calls. After a manual variant review, we estimated a false call rate of 5% (Supplementary Figure S4). Only one gene, *TP53*, the most commonly mutated gene in cancer, including HNSCC (16), was recurrently mutated with 5/6 of FOSCC samples containing a missense SNV at different positions in the DNA-binding domain (Figure 2).

Since TMB has emerged as a predictive biomarker of human patient stratification toward immunotherapy for some cancer types we evaluated TMB in FOSCC. Using our small cohort, the estimated TMB mean for each tumor was 3.7 with a range of 1.4–8.5. This was comparable to TMB calculations in HNSCC, with most tumors falling between three and seven (23). We were not able to determine if survival or response to immunotherapy was associated with TMB score due to a lack of data and immune therapeutics specific to the cat.

### Differential gene expression, cluster analysis, and functional enrichment analysis

**Figure 3:**
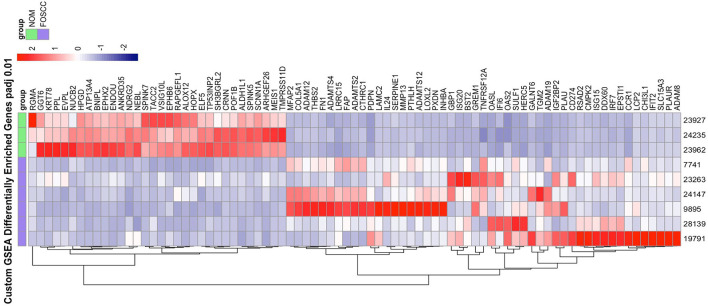
The heat map on the right illustrate the expression patterns of the top 50% leading edge genes known* to be upregulated and downregulated in human oral squamous cell carcinoma; the heat maps show clear differences between FOScc (first 6 columns) and control samples (last 3 columns), and to a much lesser extent, variation within group. NOM, normal oral mucosa; FOSCC, feline oral squamous cell carcinoma; GSEA, GENE set enrichment analysis. *Sun Y, Sang Z, Jiang Q, Ding X, Yu Y. Transcriptomic characterization of differential gene expression in oral squamous cell carcinoma: a meta-analysis of publicly available microarray data sets. Tumor Biol. 2016.

Of the 19,588 protein-coding genes annotated in Felis_catus_9.0 (Ensembl release 105), we found 2,372 differentially expressed genes (DEG; *p* < 0.05) in FOSCC (*n* = 6) when compared to normal oral mucosa (*n* = 3); of these, 1,388 genes were upregulated and 984 were downregulated (Supplementary Table S2). Principal component analysis demonstrated all samples clustered according to phenotype (Supplementary Figure S5), indicating that the primary global signal in the gene expression profiles distinguishes tumor from normal samples, regardless of anatomical location or variations in sample processing or quality. The most enriched gene sets using the GSEA MSigDB Hallmark collections are characteristic of pathways activated in cancer (Supplementary Table S3, Supplementary Figure S6). The epithelial-mesenchymal transition (EMT) gene set was the most enriched gene set, as well as signatures of pathway activation (TNFA, KRAS, IL6) and immune response was also observed in the TNFA, KRAS, hypoxia, and IL-6 signaling gene sets among others. Notably, a custom gene set consisting of the 200 most upregulated and downregulated genes in human OSCC (34) had higher normalized enrichment scores than any Hallmark gene set upregulated in human OSCC: NES 8.64, *q*-value reported as zero; downregulated in HOSCC: NES −4.07, *q*-value reported as zero; Figure 3.

### FOSCC comparison with HNSCC

We compared the somatic mutations of FOSCC genes shown to have highly recurrent mutations in HNSCC to assess similarities. Genes harboring FOSCC non-synonymous SNVs, including frameshifts, were matched to the same genes in the HNSCC Driver Database 3 (35), and OncoKB (36). *TP53* was the most recurrently mutated somatic gene in HNSCC and FOSCC, with three FOSCC samples containing a missense mutation, and one sample containing a frameshift mutation (Figure 2). A missense mutation in *KAT2B, HOX3B, MED12L, ARID1A, and KMT2D* was present in one sample (Supplementary Table S4). Samples 9895 and 23263 had the most genes implicated in HNSCC with four genes in common each (Supplementary Table S4). This data indicates some evidence for overlap in the mutational background between HNSCC and FOSCC but is very preliminary at this stage.

## Discussion

In this first study of the somatic mutations and gene expression variation present in FOSCC, we describe the similarities and differences when comparing to HNSCC, its most closely related cancer type in humans. Although FOSCC is the most common oral tumor in domestic cats, our knowledge of its genetic properties is very limited. The few characterized molecular features of FOSCC include overexpression of EGFR and perturbed *p53* expression (19, 37). When examining the commonalities of FOSCC and HNSCC, overexpression of *EGFR* in 90% of 750 HNSCC tumors sequenced, and *TP53* mutated in 41% of cases is also observed (38). We hypothesized that altered genes in FOSCC would be known to be implicated in HSCC, and would present alternate candidates for hypotheses testing of mechanisms of action (35). We find several mutated genes in common (Supplementary Table S4) the strongest candidate being *TP53*, which is the most mutated in HNSCC and all cancer types (2, 39–41). Given that *TP53* is a tumor suppressor gene and variation in *TP53* and is a predictive marker for immunotherapy in HNSCC (16) it is reasonable to hypothesize that if feline immune checkpoint drugs were available their use in the rapidly growing FOSCC could be efficacious. In agreement with our findings, *p53* mutations have been seen in other FOSCC studies (4, 37).

Immunotherapy is not an option in feline cancer treatment today but may likely be available in the future. Since TMB has been shown to be useful in some human cancer types, e.g., HHNSCC non-small cell lung cancer and melanoma (42–44), we sought to compare FOSCC to HNSCC for this metric despite our small cohort size. Recent studies on HNSCC have found that mutations in *TP53* are associated with high TMB and low overall survival rates, and coincidently HNSCC patients with high TMB have higher response rates to immune checkpoint therapy (23, 24). Moreover, HNSCC with TMB values of >5.0 is associated with poor prognosis (45, 46). Two of our FOSCC samples fall into what we suggest is a high TMB value at 8.5 and 6 and if immune checkpoint therapy was available could be evaluated for tumor control. But we recognize this is very speculative at this stage of evaluating FOSCC genetics. More FOSCC sequencing experiments to obtain better estimates of TMB are needed as well as the future availability of immune checkpoint inhibitors to improve outcomes for this very lethal cancer.

Other FOSCC recurrent or single gene mutations of interest we evaluated included *KAT2B, ARID1A, MED12L*, and *HOXB3* mostly due to their involvement in cell growth. *KAT2B* is a member of the lysine transferases that are responsible for the acetylation of a broad range of proteins that can function as tumor suppressors or oncogenes (47). HNSCC cell lines have shown universal loss of *KAT2B* (11) and HNSCC tumors show significantly lower *KAT2B* expression compared to normal tissue (48). One FOSCC sample had a missense variant in *KAT2B* (Supplementary Table S4) and it was significantly downregulated in our RNA-seq data (Supplementary Table S4) suggesting a candidate driver gene role in FOSCC. A *KMT2D* mutation was identified in only one sample but has similar epigenetic properties to *KAT2B* (49). Studies using The Cancer Genome Atlas database (TCGA) have associated mutations occurring in *KMT2D*, to the open chromatin state, thereby promoting gene expression (50, 51). Mutations in *KAT2B* and *KMT2D* may induce epigenetic changes in both HNSCC and FOSCC that could advocate for the treatment with epigenetic drug control of both (47, 51). A missense mutation in *ARID1A* was found in one sample (Supplementary Table S4), however, this gene did not show altered expression in FOSCC. *ARID1A* functions as a tumor stemness repressor by disrupting the function of the p53 or PTEN pathways (52, 53). This gene is often deleted in many human cancers (52) yet with no known role in feline cancers.

Several other candidate genes (*MED12L, HOXB3*, and *PXYLP1* with one gene mutation per sample) were found to overlap in HNSCC and FOSCC (Supplementary Table S4). Mediator Complex Subunit 12L (*MED12L*) works by activating the kinase activity of *CDK8* which regulates the growth and division of cells (54). *MED12L* is differentially overexpressed in many cancers however, the *MED12L* complex is altered in only 3.05% of HNSCC patients (55, 56). The *HOX* genes regulate a wide range of cell activity including proliferation and migration thus making their contribution to FOSCC beyond interpretation at this stage. HNSCC studies have shown an overall increased expression of all *HOX* genes, including *HOXB3* (57–59). No overlap in actionable genes mutated in FOSCC was found when searching OncoKB relative to HNSCC. We believe this is due to the low sample size of our cohort which only additional sequencing can address.

Altered gene expression is often used to discover and advance gene candidates for further study of their oncogenic roles. Transcriptional profiling showed that FOSCC is enriched with genes known to be upregulated in Human OSCC (34) suggesting conserved gene regulatory mechanisms. While these findings were predicted given the remarkable clinical, pathological, and genetic parallels between both tumors (9, 37, 60–62) they in no way confirm comparative origins or outcomes. The functional gene and canonical pathway enrichment analyses provided us further insight into the possible activation of several pathways that were seen in FOSCC that included EMT, hypoxia- and inflammation-related pathways (61–65), indicating the complementary value of gene expression analysis in this study. EMT typically involves the expression of transcription factors that can activate this cellular program (i.e., *SNAI1, TWIST1, ZEB1*, and *ZEB2*), and is characterized by the upregulation of mesenchymal-related genes (e.g., *FN1, VIM, CDH2*, and metalloproteinases) and downregulation of epithelial-related genes (i.e., *CDH1* and cytokeratins) (65–67). These studies are insightful into the aggressive biological behavior of FOSCC in part due to the activation of the EMT program. Not surprisingly activation of the hypoxia and angiogenesis pathways, as well as several inflammation pathways are predicted to be implicated in oral cancer with rapid tumor growth creating areas of ischemia and necrosis, and the production of reactive oxygen species (62). These events also induce the expression of transcription factors that can stimulate angiogenesis while simultaneously eliciting a local inflammatory response inducing expression of *PTGS2* (i.e., COX-2), with signaling *via* NF-kB, TNF, IL-6, and TGFB, all of which were found to be significantly enriched in FOSCC.

In the context of the recurrent *TP53* somatic mutations observed, it was interesting to note that the p53 pathway was not significantly activated across the set of FOSCC tumors compared to healthy controls (GSEA FDR > 0.2). This observation suggests that *TP53* mutations in FOSCC represent loss-of-function events and that the mechanisms that would normally result in p53 pathway activation are likely impaired. Consistent with cancer studies in people (68), *TP53* and relevant target genes (e.g., *CDKN1A*) known to be expressed in wild-type cells were found to be downregulated in FOSCC, while genes that would be expected to be upregulated in *TP53* mutated cells (e.g., *E2F1, MYBL2*, and *FOXM1*) were enriched in FOSCC. Overall, these findings strongly implicate *TP53* somatic mutations as driver events in FOSCC tumorigenesis.

Another aspect of FOSCC revealed by RNA-seq was the variation among tumor expression profiles. Indeed, although cluster analysis revealed a distinct molecular phenotype when compared to control samples, we observed differences among tumor samples among individual genes as well as for relevant biological pathway activity (Figure 3, Supplementary Figure S6). This tumor heterogeneity may reflect inter-individual differences (i.e., variations in tumor stage, anatomical location, breed, age, other clinical and environmental factors, etc.), and inherent variations among primary clinical samples of naturally occurring disease, which are comprised of heterogeneous cell types and frequencies and are typically collected in a clinical setting rather than under strict research protocols.

There are several limitations to this small study, with the most important factor being cohort size. Only six samples were available at the time of sequencing, therefore conclusions between potential causal variants in the cat in common with the human were found less frequently. Another limitation of the study is that healthy mucosal tissue from case animals was not available to use as matched RNA-seq controls, due to limitations of the clinical sampling protocol. Tumors also originated from different anatomical locations and were collected and processed at different times, which can contribute to transcriptional variation. Lastly, aside from *TP53*, the common mutations identified with functional effects have not been studied at length and the implication of variants in these genes are unknown. Further study is in progress in a larger cohort of cats where more significant conclusions may be drawn.

## Conclusion

The first exome resource was used to evaluate the somatic mutational landscape of FOSCC and gene expression changes were identified using RNA-seq. We observed several mutations in *TP53*, consistent with what is seen in HNSCC, and several other genes also overlapped between FOSCC and HNSCC. Our study suggests that similar genes initiate tumorigenesis in both species and perhaps FOSCC may serve as a comparative model of treatment in HNSCC. Similarities in the mechanism of FOSCC and HNSCC shown in the RNA-seq studies, such as genes implicated in inflammation further demonstrate the possible use of the domestic feline as a model for HNSCC.

## Data availability statement

The datasets presented in this study can be found in online repositories. The names of the repository/repositories and accession number(s) can be found in the article/Supplementary material.

## Author contributions

WW and JB provided samples for analysis. AR completed variant calling and analysis. ZS, OG, and SC assisted in variant calling and analysis. JKG and SP completed sample collection as well as RNA-sequencing analysis. All authors contributed to the article and approved the submitted version.
